# Identifying optimal candidates for induction chemotherapy among stage II–IVa nasopharyngeal carcinoma based on pretreatment Epstein–Barr virus DNA and nodal maximal standard uptake values of [^18^F]‐fluorodeoxyglucose positron emission tomography

**DOI:** 10.1002/cam4.3500

**Published:** 2020-10-09

**Authors:** Hao‐Jun Xie, Yi‐Fei Yu, Xue‐Song Sun, Guo‐Dong Jia, Dong‐Hua Luo, Rui Sun, Li‐Ting Liu, Shan‐Shan Guo, Sai‐Lan Liu, Qiu‐Yan Chen, Lin‐Quan Tang, Hai‐Qiang Mai

**Affiliations:** ^1^ Sun Yat‐sen University Cancer Center State Key Laboratory of Oncology in South China Collaborative Innovation Center for Cancer Medicine Guangdong Key Laboratory of Nasopharyngeal Carcinoma Diagnosis and Therapy Guangzhou P. R. China; ^2^ Department of Nasopharyngeal Carcinoma Sun Yat‐sen University Cancer Center Guangzhou P. R. China; ^3^ Department of Organ Transplant Center The First Affiliated Hospital Sun Yat‐sen University Guangzhou P. R. China

**Keywords:** Epstein–Barr virus (EBV) DNA, induction chemotherapy, nasopharyngeal carcinoma, survival, SUVmax

## Abstract

**Objective:**

This study aimed to select optimal candidates benefiting from the addition of induction chemotherapy (IC) to concurrent chemoradiotherapy (CCRT) in stage II–IVa nasopharyngeal carcinoma (NPC) based on Epstein–Barr virus (EBV) DNA and nodal maximal standardized uptake values (SUVmax‐N) of [^18^F]‐fluorodeoxyglucose positron emission tomography.

**Patients and materials:**

A total of 679 patients diagnosed with stage II–IVa (except N0) NPC were retrospectively included in this study. Overall survival was the primary endpoint. Survival differences between different groups were compared using the log‐rank test. The hazard ratio (HR) and 95% confidence interval (CI) were calculated using a multivariable Cox proportional hazards model.

**Results:**

Both high levels of EBV DNA (>1500 copies/mL) and SUVmax‐N (>12.3) indicated worse survival conditions. All patients were divided into low‐ and high‐risk groups based on these two biomarkers. The risk group was an independent prognostic factor in OS, progression‐free survival (PFS), and distant metastasis‐free survival (DMFS) (all *p*‐values<0.05). The addition of IC to CCRT was associated with survival improvement in OS, PFS, and DMFS in high‐risk patients, while no survival difference was found between CCRT and IC+CCRT in low‐risk patients.

**Conclusions:**

Our study can help clinicians select stage II–IVa NPC patients who benefit from IC, which is important in guiding individual treatment.

## Graphical abstract

Our study has demonstrated that EBV DNA and SUVmax‐N could serve as biomarkers to stratify NPC patients and guide physicians to select those who benefit from IC. Our findings provide important information for personalizing NPC treatment. Prospective clinical trials are warranted to evaluate the results of this study.

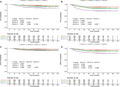

## 1 INTRODUCTION

Nasopharyngeal carcinoma (NPC) is a unique malignancy that differs from other head and neck cancer in terms of geographical distribution, treatment method, and prognosis.^1^ In 2018, approximately 12,900 new cases of NPC were reported, accounting for 0.7% of all cancers, among which greater than 70% cases occurred in east and southeast Asia.^2^ Radiotherapy (RT) is the mainstay treatment modality for NPC, as it is highly sensitive to radiation.^3^ For locoregionally advanced NPC (LANPC), previous studies have demonstrated the superiority of concurrent chemoradiotherapy (CCRT) in survival benefit compared with RT alone.^4^, ^5^, ^6^

Recently, an increasing number of studies have investigated the role of induction chemotherapy (IC). For LANPC, randomized Phase III trials demonstrated that the addition of IC before standard CCRT was associated with survival improvement.^7^, ^8^ However, this aggressive treatment method is also accompanied by more serious toxicity.^7^ A previous study verified that TNM stage is insufficient to reflect the tumor burden and predict survival accurately for NPC patients.^9^ Therefore, it is necessary to combine the extent of tumor invasion and effective biomarkers to divide patients into different risk levels and identify optimal candidates for the IC treatment.

Plasma Epstein–Barr virus (EBV) DNA levels are useful in the detection, monitoring, and prognostic prediction of both nonmetastatic and metastatic NPC and are regarded as the most important biomarker.^10^ Previous studies suggested that the maximal standardized uptake value (SUVmax) of [18F]‐fluorodeoxyglucose positron emission tomography (PET) of cervical lymph node (SUVmax‐N) was also an important prognostic factor in NPC.^11^, ^12^ According to NCCN guidelines, IC was recommended for stage II–IVa NPC.^13^ In this study, we explored the prognostic value of EBV DNA and SUVmax‐N among stage II–IVa patients and then investigated the role of IC in patients with different risk levels with the aim of selecting patients who can truly benefit from IC.

## 2 MATERIALS AND METHODS

### 2.1 Patients

Between January 2008 and December 2013, 679 NPC patients were screened from the database of the Sun Yat‐Sen University Cancer Center (SYSUCC). The inclusion criteria were as follows: (1) pathological diagnosis of NPC; (2) stage II–IVa (except N0) based on the 8th edition of the AJCC/UICC staging system (3) assessed by PET/computed tomography (CT); (4) available pretreatment EBV DNA data; (5) use of platinum‐based CCRT with or without IC; (6) no adjuvant chemotherapy application (7) Karnofsky performance score >70; (8) normal liver and kidney functions; (9) without lactation and pregnancy; (10) without other malignant diseases. Clinical research ethics committee at SYSUCC approved this study.

### 2.2 Diagnosis and treatment

All the patients underwent complete pretreatment evaluations at admission and were treated based on the principles of SYSUCC. All the patients received the examination of plasma EBV DNA level. The detailed information on method of plasma EBV DNA quantification and treatment is available in Supplementary Materials.

### 2.3 Follow‐up

Patients were examined every three months for two years after treatment, and then every six months until death. The routine follow‐up evaluation included physical examination, nasopharyngeal fiber optic endoscopy, MRI/CT of the head and neck, chest radiography/CT, abnormal ultrasound/CT, bone scan, and PET/CT if necessary. The primary endpoint of our study was overall survival (OS), which was defined as the time from the date of diagnosis to the date of death from any cause. The following survival outcomes were secondary endpoints: progression‐free survival (PFS) was calculated from the date of diagnosis to the date of disease progression or death for any reason; relapse‐free survival (LRFS) and distant metastasis‐free survival (DMFS) were calculated from the date of diagnosis to the date of locoregional failure and distant metastasis, respectively.

### 2.4 Statistical analyses

The detailed information of statistical analyses is available in Supplementary Materials.

## 3 RESULTS

### 3.1 Patients’ characteristic and survival

From 2008 to 2013, 679 stage II–IVa NPC patients were retrospectively involved in this study. The median age of our cohort was 46 years; the male to female ratio was 3.5:1. EBV DNA and SUVmax‐N were transferred to categorical variables for further analysis and were derived from the published cut‐off point (1500 copies/mL) and ROC curve, respectively. The median concentration of EBV DNA was 3.55 × 10^3^ copies/mL (range 0–6.9 × 10^6^ copies/mL). A total of 420 patients had EBV DNA levels greater than 1500 copies/mL. The SUVmax cut‐off point was 12.3 for OS (AUC [area under the curve] = 0.607, *p* = 0.006) (Figure S1). There were 421 patients (62.0%) assigned to the lower SUVmax‐N group (≤12.3), and 258 patients (38.0%) had a greater SUVmax‐N value. The patients’ characteristics are presented in Table 1.

**Table 1:** Baseline characteristics of patients

Characteristic	Number of patients, n (%)
Total	679
Age, y	
≤46	346 (51.0%)
>46	333 (49.0%)
Gender	
Female	150 (22.1%)
Male	529 (77.9%)
Smoking history	
No	404 (59.5%)
Yes	275 (40.5%)
Family history of NPC	
No	617 (90.9%)
Yes	62 (9.1%)
Overall stage^a^	
II	42 (6.2%)
III	382 (56.3%)
IV	255 (37.6%)
T stage^a^	
T1	36 (5.3%)
T2	101 (14.9%)
T3	370 (54.5%)
T4	172 (25.3%)
N stage^a^	
N1	282 (41.5%)
N2	286 (42.1%)
N3	111 (16.3%)
LDH level	
≤245 U/L	630 (92.8%)
>245 U/L	49 (7.2%)
SUVmax‐N	
≤12.3	421 (62.0%)
>12.3	258 (38.0%)
EBV DNA level	
≤1500 copies/mL	259 (38.1%)
1500–4000 copies/mL	96 (14.1%)
EBV>4000 copies/ml	324 (47.7%)
Treatment method	
CCRT alone	366 (53.9%)
CCRT+IC	313 (46.1%)
IC regimen*	
TPF	157 (50.2%)
PF	68 (21.7%)
TP	54 (17.3%)
GP	34 (10.9%)
IC cycle*	
2 cycles	164 (52.4%)
3 cycles	111 (35.5%)
4 cycles	38 (12.1%)

Abbreviations: EBV, Epstein–Barr virus; GP, cisplatin plus gemcitabine; IC, induction chemotherapy; LDH, lactate dehydrogenase, CCRT, concurrent chemoradiotherapy; PF, cisplatin plus 5‐fluorouracil; TP, cisplatin plus docetaxel; TPF cisplatin plus docetaxel plus 5‐fluorouracil.

*p*‐values were calculated using a χ^2^ test.

^a^ According to the 8th edition of the UICC/AJCC staging system.

* Only patients treated with IC+CCRT were analyzed.

The median follow‐up time was 71.5 months (interquartile range [IQR]: 60.7–83.1). A total of 36 patients (4.7%) lost to follow‐up within 3 years. The 3‐year OS, PFS, LRFS, and DMFS in our cohort were 93.6%, 85.4%, 94.8%, and 89.8%, respectively.

### 3.2 Risk stratification

**Figure 1:**
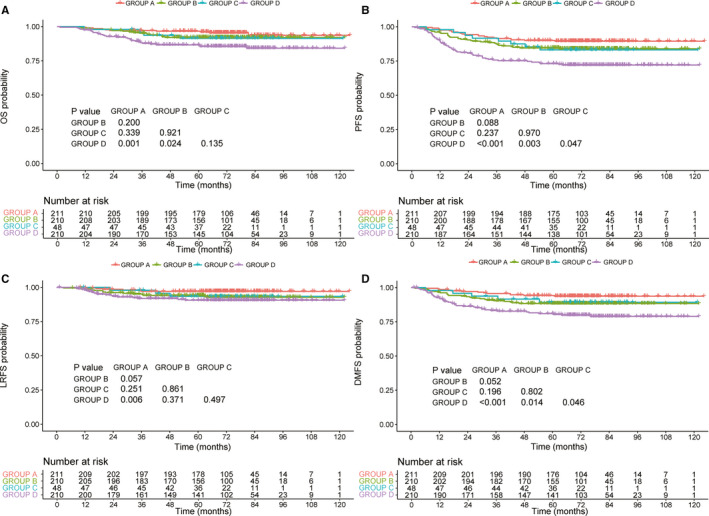
Kaplan–Meier in 679 patients based on a combination of EBV DNA and SUVmax‐N: overall survival (A), progression‐free survival (B), locoregional relapse‐free survival (C), and distant metastasis‐free survival (D)

As shown in Figure S2, patients with higher pre‐EBV DNA levels suffered worse survival conditions in all endpoints (all *p*‐values<0.05). Similarly, a higher SUVmax‐N (>12.3) was also associated with significantly lower OS, PFS, LRFS, and DMFS (Figure S3). Based on these two prognostic biomarkers, we subdivided all patients into four subgroups: group A, SUVmax‐N ≤ 12.3 and EBV DNA≤1500; group B, SUVmax‐N ≤ 12.3 and EBV DNA>1500; group C, SUVmax‐N > 12.3 and EBV DNA≤1500; group D, SUVmax‐N > 12.3 and EBV DNA>1500. Compared with other three groups, group D had the lowest PFS and DMFS (all *p*‐values<0.05). Besides, we can observe the significant difference in OS between group D and group A/B (all *p*‐values<0.05). Further pairwise comparisons revealed no significant difference in OS, PFS, and DMFS among groups A–C (all *p*‐values>0.05). In terms of LRFS, a significant difference was only observed between group A and group D (Figure 1). Therefore, we combined groups A–C into the low‐risk group, and group D served as the high‐risk group. There were a total of 469 and 210 patients in each subgroup, respectively.

**Figure 2:**
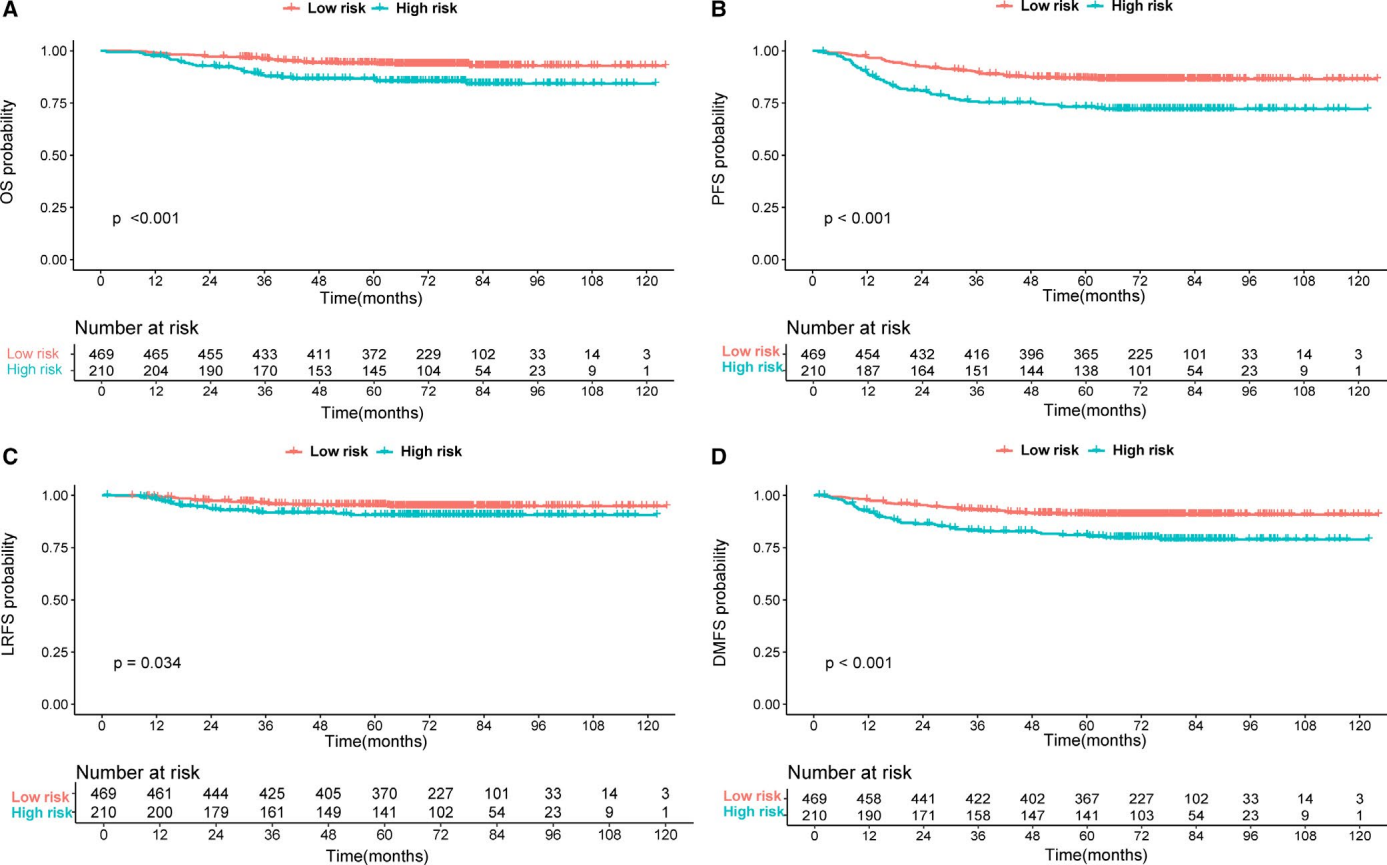
Kaplan–Meier in 679 patients grouped by risk stratification: overall survival (A), progression‐free survival (B), locoregional relapse‐free survival (C), and distant metastasis‐free survival (D)

The 3‐year OS, PFS, LRFS, and DMFS rates of patients in low‐ and high‐risk groups were 95.9% versus 87.7%, 89.7% versus 75.2%, 96.3% versus 91.9%, and 92.9% versus 83.2%, respectively (all *p*‐values<0.05)(Figure 2). After adjusting for other variables, the risk group was also identified as an independent prognostic factor for OS, PFS, and DMFS in multivariate analysis (all *p*‐values<0.05).

### 3.3 Treatment outcome for patients in different risk groups

Before radical CCRT, a total of 313 patients (46.1%) received IC, with 164 patients receiving two cycles, 111 and 38 patients receiving three cycles, and four cycles, respectively. The number of patients in each IC regimen was 157 (cisplatin plus docetaxel plus 5‐fluorouracil), 68 (cisplatin plus 5‐fluorouracil), 54 (cisplatin plus docetaxel), and 34 (cisplatin plus gemcitabine), respectively (Table 1). In multivariate analysis, the application of IC was associated with a lower risk of distant metastasis (*p* = 0.046) (Table 2). However, there was no association between different IC regimens and survival outcomes in all endpoints (Figure S4). The addition of IC increased the risk of grade 3–4 leukopenia (32.8 vs 16.3%; *p* < 0.001) and neutropenia (39.5 vs 11.6%; *p* < 0.001). Details of other acute toxicity by treatment groups are presented in Table S2.

**Table 2:** Univariable and multivariable analysis of OS, PFS, LRFS, and DMFS

Characteristic	Univariable analysis			Multivariable analysis		
	HR	95% CI	*p*‐value	HR	95% CI	*p* ‐value
Overall Survival						
Age	1.592	0.950‐2.669	0.078	1.559	0.930‐2.614	0.092
Gender	1.296	0.674‐2.493	0.437			
Smoking	1.387	0.836‐2.303	0.205			
Family history of NPC	1.116	0.480‐2.595	0.798			
Overall stage	1.739	1.048‐2.885	0.032			
T stage	1.499	0.877‐2.565	0.139			
N stage	4.892	2.324‐10.298	<0.001	4.540	2.149‐9.592	<0.001
LDH level	3.085	1.604‐5.934	0.001	2.422	1.255‐4.674	0.008
Risk group	2.404	1.449‐3.988	0.001	2.020	1.045‐3.522	0.032
Treatment method	0.883	0.530‐1.471	0.632	0.629	0.371‐1.067	0.085
Progression‐free survival						
Age	1.427	0.994‐2.050	0.054			
Gender	0.990	0.645‐1.519	0.963			
Smoking	1.056	0.734‐1.518	0.770			
Family history of NPC	1.221	0.656‐2.171	0.497			
Overall stage	1.823	1.274‐2.607	0.001	1.488	1.029‐2.152	0.047
T stage	1.289	0.872‐1.904	0.203			
N stage	2.127	1.419‐3.188	<0.001	1.654	1.079‐2.535	0.021
LDH level	1.997	1.163‐3.430	0.012			
Risk group	2.298	1.606‐3.289	<0.001	1.827	1.248‐2.675	0.002
Treatment method	0.966	0.675‐1.384	0.851	0.711	0.488‐1.037	0.077
Loco‐regional relapse‐free survival						
Age	1.212	0.656‐2.240	0.539			
Gender	1.189	0.549‐2.574	0.661			
Smoking	0.937	0.500‐1.754	0.838			
Family history of NPC	1.745	0.734‐4.148	0.208			
Overall stage	3.052	1.616‐5.763	0.001	3.052	1.616‐5.763	0.001
T stage	1.912	1.021‐3.582	0.043			
N stage	1.065	0.572‐1.982	0.843			
LDH level	1.486	0.530‐4.168	0.452			
Risk group	1.925	1.038‐3.567	0.038			
Treatment method	1.826	0.975‐3.420	0.060			
Distant metastasis‐free survival						
Age	1.381	0.895‐2.132	0.145			
Gender	1.038	0.616‐1.750	0.889			
Smoking	1.029	0.664‐1.593	0.899			
Family history of NPC	1.062	0.513‐2.202	0.871			
Overall stage	1.396	0.905‐2.152	0.131			
T stage	0.951	0.575‐1.573	0.846			
N stage	3.481	1.990‐6.088	<0.001	2.902	1.629‐5.169	<0.001
LDH level	2.146	1.138‐4.048	0.018			
Risk group	2.436	1.584‐3.747	<0.001	1.855	1.189‐2.893	0.006
Treatment method	0.825	0.534‐1.275	0.386	0.630	0.400‐0.992	0.046

A Cox proportional hazard model was used to perform multivariate analyses. All variables were transformed into categorical variables. HRs were calculated for age (years) (>46 vs ≤46); gender (male vs female); smoking (yes vs no); family history of NPC (yes vs no); overall stage (IV vs II–III); T stage (T3‐4 vs T1‐2); N stage (N2‐3 vs N0‐1); LDH level (>245 U/L vs ≤245 U/L); risk group (high‐risk vs low‐risk) and treatment method (CCRT+IC vs CCRT).

Abbreviations: CI, confidence interval; HR, hazard ratio; LDH, lactate dehydrogenase; NPC, nasopharyngeal carcinoma.

**Figure 3:**
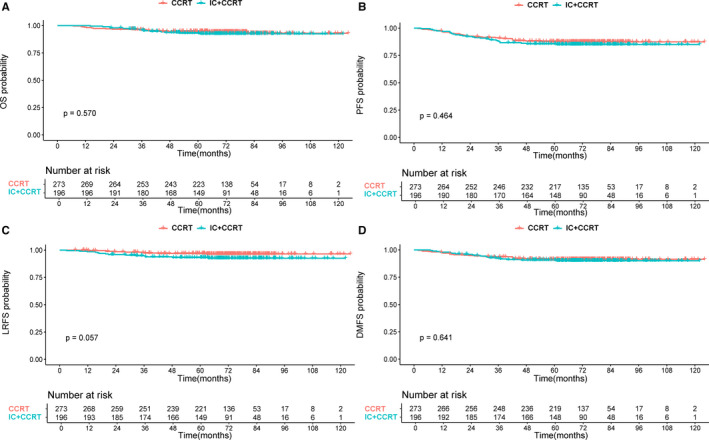
Kaplan–Meier in 469 low‐risk patients grouped by treatment methods (CCRT alone or IC+CCRT): overall survival (A), progression‐free survival (B), locoregional relapse‐free survival (C), and distant metastasis‐free survival (D

**Figure 4:**
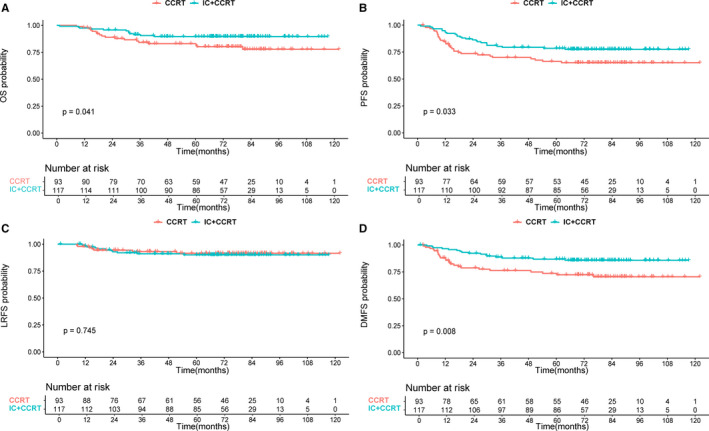
Kaplan–Meier in 210 high‐risk patients grouped by treatment methods (CCRT alone or IC+CCRT): overall survival (A), progression‐free survival (B), locoregional relapse‐free survival (C), and distant metastasis‐free survival (D)

We further analyzed the effect of different treatment methods (IC+CCRT or CCRT alone) on prognosis among patients with different risk levels. The characteristics of patients in different risk subgroups are shown in Table S1. Interestingly, we found that the role of IC was different between low‐ and high‐risk groups. In the low‐risk group, patients treated with IC plus CCRT had similar survival outcomes for OS, PFS, LRFS, and DMFS compared with those treated with CCRT alone (all *p*‐values>0.05) (Figure 3). However, in the high‐risk group, the addition of IC achieved significant improvements in OS, PFS, and DMFS (3‐year OS: 90.5% vs 84.2%; *p* = 0.041; 3‐year PFS: 80.4% vs 70.0%; *p* = 0.033; 3‐year DMFS: 87.8% vs 76.2%; *p* = 0.008) (Figure 4). Stratified multivariate analysis also demonstrated IC application was an independent protective factor for OS (HR: 0.453, 95% CI: 0.218‐0.941; *p* = 0.034), PFS (HR: 0.571, 95% CI: 0.339‐0.962; *p* = 0.035) and DMFS (HR: 0.436, 95% CI: 0.233‐0.817; *p* = 0.010) in high‐risk patients but not associated with better prognosis in low‐risk patients (Tables 3 and 4).

**Table 3:** Univariable and multivariable analysis of OS, PFS, LRFS, and DMFS for low‐risk group

Characteristic	Univariable analysis			Multivariable analysis		
	HR	95% CI	*p*‐value	HR	95% CI	*p* ‐value
Overall survival						
Age	1.451	0.705‐2.987	0.313			
Gender	4.208	1.002‐17.669	0.050	4.476	1.059‐18.925	0.042
Smoking	2.531	1.204‐5.319	0.014			
Family history of NPC	1.734	0.605‐4.969	0.305			
Overall stage	2.284	1.116‐4.674	0.024	1.896	0.922‐3.903	0.082
T stage	1.659	0.776‐3.544	0.191			
N stage	4.354	1.779‐10.655	0.001	3.705	1.499‐9.159	0.005
EBV DNA	1.466	0.716‐3.005	0.296			
SUVmax‐N	1.328	0.463‐3.805	0.598			
LDH level	4.592	1.601‐13.169	0.005	3.967	1.363‐11.547	0.011
Treatment method	1.231	0.601‐2.522	0.571			
Progression‐free survival						
Age	1.577	0.955‐2.604	0.075			
Gender	1.392	0.726‐2.668	0.319			
Smoking	1.243	0.758‐2.041	0.389			
Family history of NPC	1.204	0.519‐2.792	0.666			
Overall stage	1.832	1.114‐3.012	0.017	1.733	1.052‐2.854	0.031
T stage	1.321	0.765‐2.282	0.318			
N stage	1.926	1.153‐3.217	0.012	1.836	1.097‐3.073	0.021
EBV DNA	1.433	0.874‐2/350	0.154			
SUVmax‐N	1.256	0.598‐2.637	0.547			
LDH level	2.713	1.088‐6.765	0.032			
Treatment method	1.203	0.733‐1.975	0.464			
Loco‐regional relapse‐free survival						
Age	1.433	0.628‐3.269	0.392			
Gender	1.065	0.395‐2.869	0.901			
Smoking	0.934	0.404‐2.159	0.874			
Family history of NPC	1.074	0.252‐4.582	0.923			
Overall stage	4.310	1.827‐10.166	0.001	4.310	1.827‐10.166	0.001
T stage	3.045	1.343‐6.901	0.008			
N stage	0.831	0.364‐1.896	0.660			
EBV DNA	2.014	0.872‐4.655	0.101			
SUVmax‐N	1.290	0.383‐4.343	0.680			
LDH level	1.343	0.181‐9.966	0.773			
Treatment method	2.209	0.956‐5.104	0.064			
Distant metastasis‐free survival						
Age	1.628	0.879‐3.015	0.121			
Gender	1.764	0.743‐4.187	0.198			
Smoking	1.206	0.657‐2.215	0.545			
Family history of NPC	1.536	0.604‐3.907	0.368			
Overall stage	1.131	0.596‐2.149	0.706			
T stage	0.655	0.291‐1.475	0.307			
N stage	4.048	1.937‐8.461	<0.001	3.936	1.881‐8.236	<0.001
EBV DNA	1.719	0.933‐3.168	0.082			
SUVmax‐N	1.175	0.462‐2.990	0.735			
LDH level	3.361	1.199‐9.422	0.021	2.886	1.028‐8.105	0.044
Treatment method	1.155	0.629‐2.121	0.641			

A Cox proportional hazard model was used to perform multivariate analyses. All variables were transformed into categorical variables. HRs were calculated for age (years) (>46 vs ≤46); gender (male vs female); smoking (yes vs no); family history of NPC (yes vs no); overall stage (IV vs II–III); T stage (T3‐4 vs T1‐2); N stage (N2‐3 vs N0‐1); EBV DNA (>1500 copies/ml vs ≤1500 copies/ml); SUVmax‐N (>12.3 vs ≤12.3); LDH level (>245 U/L vs ≤245 U/L) and treatment method (CCRT+IC vs CCRT).

Abbreviations: CI, confidence interval; HR, hazard ratio; LDH, lactate dehydrogenase; NPC, nasopharyngeal carcinoma.

**Table 4:** Univariable and multivariable analysis of OS, PFS, LRFS, and DMFS for high‐risk group

Characteristic	Univariable analysis			Multivariable analysis		
	HR	95% CI	*p*‐value	HR	95% CI	*p* ‐value
Overall survival						
Age	1.659	0.789‐3.486	0.182			
Gender	0.638	0.292‐1.394	0.260			
Smoking	0.761	0.356‐1.626	0.481			
Family history of NPC	0.564	0.134‐2.368	0.434			
Overall stage	0.967	0.473‐1.978	0.927			
T stage	1.203	0.563‐2.569	0.634			
N stage	3.780	0.900‐15.870	0.069	4.141	0.984‐17.422	0.053
LDH level	1.700	0.730‐3.963	0.219			
Treatment method	0.487	0.234‐1.011	0.054	0.453	0.218‐0.941	0.034
Progression‐free survival						
Age	1.188	0.704‐2.004	0.520			
Gender	0.696	0.390‐1.240	0.218			
Smoking	0.889	0.519‐1.522	0.668			
Family history of NPC	1.059	0.480‐2.336	0.887			
Overall stage	1.324	0.783‐2.241	0.295			
T stage	1.110	0.635‐1.940	0.715			
N stage	1.423	0.698‐2.900	0.332			
LDH level	1.148	0.580‐2.273	0.691			
Treatment method	0.571	0.339‐0.962	0.035	0.571	0.339‐0.962	0.035
Loco‐regional relapse‐free survival						
Age	0.925	0.367‐2.331	0.869			
Gender	1.358	0.393‐4.691	0.628			
Smoking	0.948	0.368‐2.447	0.913			
Family history of NPC	2.315	0.762‐7.034	0.139			
Overall stage	1.537	0.596‐3.965	0.374			
T stage	0.906	0.323‐2.542	0.851			
N stage	0.945	0.311‐2.871	0.920			
LDH level	1.120	0.324‐3.868	0.858			
Treatment method	1.170	0.454‐3.020	0.745			
Distant metastasis‐free survival						
Age	1.088	0.589‐2.011	0.787			
Gender	0.653	0.333‐1.280	0.215			
Smoking	0.885	0.469‐1.671	0.707			
Family history of NPC	0.590	0.182‐1.910	0.378			
Overall stage	1.225	0.661‐2.271	0.519			
T stage	1.134	0.587‐2.190	0.708			
N stage	1.538	0.647‐3.656	0.330			
LDH level	1.127	0.500‐2.543	0.773			
Treatment method	0.436	0.233‐0.817	0.010	0.436	0.233‐0.817	0.010

A Cox proportional hazard model was used to perform multivariate analyses. All variables were transformed into categorical variables. HRs were calculated for age (years) (>46 vs ≤46); gender (male vs female); smoking (yes vs no); family history of NPC (yes vs no); overall stage (IV vs II–III); T stage (T3‐4 vs T1‐2); N stage (N2‐3 vs N0‐1); LDH level (>245 U/L vs ≤245 U/L) and treatment method (CCRT+IC vs CCRT).

Abbreviations: CI, confidence interval; HR, hazard ratio; LDH, lactate dehydrogenase; NPC, nasopharyngeal carcinoma.

## 4 DISCUSSION

In the current study, we retrospectively examined 679 stage II–IVa (except N0) NPC patients who underwent PET‐CT examinations and verified the prognostic value of SUVmax‐N and EBV DNA. According to these two biomarkers, we divided patients into low‐ and high‐risk groups. Comparing the survival conditions between patients receiving IC+CCRT and CCRT alone, we found that the addition of IC could only benefit patients in the high‐risk group, whereas the benefit was not consistent in the low‐risk group.

For locoregionally advanced NPC, CCRT is established as the standard treatment method according to previous clinical trials.^4^, ^5^, ^6^ Recently, an increasing number of scholars have paid attention to the treatment value of IC in LANPC. Based on the results of a recent phase III trial, Ma et al. and colleagues verified that the application of IC before CCRT could further improve DMFS and OS compared with CCRT alone for III–IV LANPC (except T3‐4 N0).^7^, ^8^ In addition, the results of a network meta‐analysis, which concluded that the addition of IC to CCRT achieved the highest effect on distant control, also affirmed the curative effect of IC.^14^ However, the use of induction chemotherapy also increased the treatment‐related toxicity and the financial burden.^7^, ^8^ Thus, it is necessary to identify proper candidates who could benefit from the addition of IC. Currently, the selection of patients suitable for IC mainly depends on TNM stage, which indicates the anatomical extent of tumor. According to NCCN guidelines, all stage II–IVa NPC patients were eligible to receive IC.^13^ However, we should note that cancer is an individual disease with biological heterogeneity.^9^ Thus, it is not rigorous to decide patients who might benefit from IC exclusively based on TNM stage.

Plasma EBV DNA level represents an important biomarker for the clinical management of NPC.^10^, ^15^, ^16^, ^17^, ^18^ Lin et al. reported that patients with pre‐EBV DNA levels of greater than 1500 copies/mL faced a higher risk of treatment failure compared with other patients.^10^ Consistent with this study, we choose 1500 copies/mL as the cutoff value of EBV DNA. With the development of imaging technology, PET‐CT has been increasingly applied in the diagnosis of NPC, especially in the early detection of distant lesions.^11^, ^19^ In terms of the prognostic value of SUVmax, previous studies showed that SUVmax‐N was an independent prognostic factor both in LANPC and metastatic NPC, while the SUVmax of the primary tumor was not.^11^, ^12^ To select patients with higher tumor burden, which indicates that more aggressive treatment is necessary, we combined these two biomarkers to divide patients into different risk groups and investigated the role of IC among them.

The current study confirmed the results of past studies that demonstrated that SUVmax‐N and EBV DNA in PET are prognostic factors for NPC patients. Based on these two factors, all patients were divided into four groups. After pairwise survival comparisons, patients with SUVmax‐N > 12.3 and EBV DNA > 1500 copies/mL were identified as high‐risk patients. When the role of IC was investigated in patients with different risk levels, we found that only high‐risk patients could benefit from the addition of IC. Although some researchers tried to identify the LANPC beneficial from IC therapy based on different prognostic factors,^20^, ^21^ the current study first verified that SUVmax‐N and EBV DNA could serve as a supplement to TNM staging to select candidates. Given that IC could result in excessive treatment toxicities, these biomarkers provided important information for individual treatment. For stage II–IVa NPC, clinicians can perform risk stratification of these patients at admission based on their SUVmax‐N and EBV DNA levels and then screen high‐risk patients for IC before radiotherapy. Low‐risk subgroups need further investigations in future IC‐related studies. The reason for the different impacts of IC on patient outcomes could be explained as follows. Higher EBV DNA and SUVmax levels are associated with tumor burden, which indicates a higher risk of distant metastasis after treatment.^9^, ^22^ Thus, a more aggressive treatment was necessary to reduce tumor burden, which helps to further improve distant control and subsequent survival. In addition, patients with high‐risk levels might develop micrometastases at diagnosis that could not be detected by existing imaging technology. The administration of IC is intended to eradicate micrometastases earlier and may be associated with more potential benefit for high‐risk patients.

There are several limitations to the current study. First, this is a retrospective study, and inherent selective bias was unavoidable. Second, there were physician biases contributing to treatment heterogeneity in IC application, regimen and duration of IC. Third, all patients involved in this study were from one treatment center, and WHO III was the main pathological type. Finally, no consensus was reached on the measure of FDG uptake. Given that SUVmax‐N is associated with image noise, the SUVmax‐N cutoff value in current study should be validated in another center.

## 5 CONCLUSION

Our study showed that EBV‐DNA and SUVmax‐N could be used as biomarkers for risk stratification of NPC patients, and more importantly, guide physicians to select those benefiting from IC. Our results provide important information for individualized NPC treatment.

## CONFLICT OF INTEREST

The authors declare no competing interests.

## AUTHOR CONTRIBUTIONS

Study concepts: Hai‐Qiang Mai, Lin‐Quan Tang, Qiu‐Yan Chen. Study design: Hao‐Jun Xie, Yi‐Fei Yu, Xue‐Song Sun, Guo‐Dong Jia, Dong‐Hua Luo, Rui Sun. Data acquisition: Hao‐Jun Xie, Yi‐Fei Yu, Xue‐Song Sun, Guo‐Dong Jia, Dong‐Hua Luo,Rui Sun, Li‐Ting Liu, Shan‐Shan Guo, Sai‐Lan Liu. Quality control of data and algorithms: Hao‐Jun Xie, Yi‐Fei Yu, Xue‐Song Sun, Dong‐Hua Luo, Guo‐Dong Jia. Data analysis and interpretation: Hao‐Jun Xie, Yi‐Fei Yu, Xue‐Song Sun, Rui Sun, Dong‐Hua Luo, Guo‐Dong Jia, Li‐Ting Liu, Shan‐Shan Guo, Sai‐Lan Liu, Hai‐Qiang Mai, Lin‐Quan Tang, Qiu‐Yan Chen. Statistical analysis: Hao‐Jun Xie, Yi‐Fei Yu, Xue‐Song Sun, Dong‐Hua Luo, Rui Sun. Manuscript preparation: Hao‐Jun Xie, Yi‐Fei Yu, Xue‐Song Sun, Guo‐Dong Jia, Rui Sun, Dong‐Hua Luo, Li‐Ting Liu, Shan‐Shan Guo, Sai‐Lan Liu, Hai‐Qiang Mai, Lin‐Quan Tang, Qiu‐Yan Chen. Manuscript editing: Hao‐Jun Xie, Yi‐Fei Yu, Xue‐Song Sun, Guo‐Dong Jia. Manuscript review: Hai‐Qiang Mai, Lin‐Quan Tang, Qiu‐Yan Chen.

## ETHICS APPROVAL AND CONSENT TO PARTICIPATE

This retrospective study was approved by the Clinical Research Committee of Sun Yat‐Sen University Cancer Center. Patients were required to provide written informed consent before enrolling in the study.

## Supporting information

Supplementary Material

Fig S1

Fig S2

Fig S3

Fig S4

Table S1

Table S2

## Data Availability

The datasets used and/or analyzed in this study can be obtained from the authors as reasonably required.
