# Supplementary material for: Identifying optimal candidates for induction chemotherapy among stage II–IVa nasopharyngeal carcinoma based on pretreatment Epstein–Barr virus DNA and nodal maximal standard uptake values of [18F]‐fluorodeoxyglucose positron emission tomography
Source: Cancer Med. 2020 Oct 9;9(23):8852–63. doi: 10.1002/cam4.3500 (PMC7724500; doi:10.1002/cam4.3500)
Supplement: Supplementary file 1 — Supplementary Material [file CAM4-9-8852-s001.docx]

***Diagnosis and treatment***

The conventional work-up includes a complete physical examination, endoscopic examination of the nasopharynx, magnetic resonance imaging (MRI) / CT of the head and neck and hematological examination, including hematologic and biochemistry profile. Plasma EBV DNA level was measured by real-time quantitative polymerase chain reaction (PCR) before the initiation of treatment. Before PET-CT images were obtained, all patients fasted for at least 6 hours. Patients with fasting blood glucose greater than 200 mg/dl were excluded. PET/CT imaging was performed using an integrated PET/CT system (Discovery ST 16; GE Healthcare, Little Chalfont, United Kingdom) based on published guidelines [1]. All patients were restaged based on the 8th edition of the American Joint Committee on Cancer (AJCC) staging system.

Three hundreds and forty-two (44.2%) patients received IC before CCRT. The common IC regimens were PF: cisplatin (80 mg/m^2^) with 5-fluorouracil (800 mg/m^2^/day over 120 h), TP: cisplatin (80 mg/m^2^) with docetaxel (80 mg/m^2^), TPF: cisplatin (60 mg/m^2^) with 5-fluorouracil (600 mg/: m^2^ over 120 h) plus docetaxel (60 mg/m^2^), and GP: cisplatin (80 mg/m^2^) with gemcitabine (1000 mg/m^2^). All IC regimens were administered at 3-week intervals for two or three cycles. All patients were treated with radical intensity-modulated radiotherapy (IMRT), which was designed based on previous studies [2, 3]. The concurrent chemotherapy regimens consisted of cisplatin/nedaplatin (80 or 100 mg/m^2^) given in weeks 1, 4, and 7 of radiotherapy or cisplatin/nedaplatin (40 mg/m^2^) given weekly during radiotherapy beginning on the first day of radiotherapy.

***Quantification of plasma EBV DNA levels***

DNA Extraction from Plasma Samples

Samples of peripheral blood (5 mL) were collected in an ethylenediamine tetraacetic acid tube from all participants and were centrifuged at ×1600g for isolation of plasma. Plasma samples were transferred carefully into plain polypropylene tubes for storage at﹣80 °C until further processing. DNA from plasma samples was extracted with the QIAamp Blood Kit (Qiagen, Hilden, Germany) using the blood and body fluid protocol, as recommended by the manufacturer. In total, 500 –1000µL of each plasma sample were used for DNA extraction per column, with a final elution volume of 50µL from the extraction column. The exact amounts were documented for the calculation of the target DNA concentration.

Real-Time Quantitative EBV DNA PCR

The real-time quantitative PCR system was developed for EBV DNA detection toward the BamHI-W region. The system consisted of the amplification primers W-44F (5’-AGT CTC TGC CTC CAG GCA-3’) and W-119R (5’-ACA GAG GGC CTG TCC ACC G-3’) and the dual-labeled fluorescent probe W-67T (5’- [FAM] CAC TGT CTG TAA AGT CCA GCC TCC [TAMRA]-3’). In this study, real-time quantitative PCR for the β-actin gene was used as a control for the amplifiability of plasma DNA. Theβ-actin gene primer sequence was forward, 5’-ACA GGC ACCA GGG CGT GA TGG-3’; and reverse, 5’-CTC CAT GTC GTC CCA GTT GGT-3’; and the dual-labeled fluorescent probe sequence (5’-[FAM] CAT CCT CAC CCT GAA GTA CCC CAT C [TAMRA]-3’).

***Statistical analyses***

All the statistical analyses were performed by SPSS for Mac version 21.0 (SPSS Inc., Chicago, IL). Receiver operating characteristic (ROC) curve analysis was used to determine the cut-off value for SUVmax-N, which showed the best trade-off between sensitivity and specificity. Kaplan–Meier curves were applied to present time-to-event data, and different groups were compared using log-rank tests. The patients’ baseline characteristics between different treatment groups were compared by the Pearson χ2 test. The Cox proportional-hazards model was used to calculate the hazard ratio (HR) and 95% confidence interval (CI) in multivariate analyses. The backward selection was used to test the independent significance of different factors. The p value threshold was 0·1 (p>0·1) for removing non-significant variables from the multivariate analysis, while marginally significant variables (0·05<p<0·1) remained in the final Cox model. All analyses were 2-side, and the level of significance was set at P < 0.05.

1. Delbeke D, Coleman RE, Guiberteau MJ, Brown ML, Royal HD, Siegel BA, Townsend DW, Berland LL, Parker JA, Hubner K *et al*: **Procedure guideline for tumor imaging with 18F-FDG PET/CT 1.0**. *Journal of nuclear medicine : official publication, Society of Nuclear Medicine* 2006, **47**(5):885-895.

2. Zhao C, Han F, Lu L, Huang S, Lin C, Deng X, Lu T, Cui N: **[Intensity modulated radiotherapy for local-regional advanced nasopharyngeal carcinoma]**. *Ai zheng = Aizheng = Chinese journal of cancer* 2004, **23**(11 Suppl):1532-1537.

3. Yu Z, Luo W, Zhou QC, Zhang QH, Kang DH, Liu MZ: **Impact of changing gross tumor volume delineation of intensity-modulated radiotherapy on the dose distribution and clinical treatment outcome after induction chemotherapy for the primary locoregionally advanced nasopharyngeal carcinoma**. *Ai zheng = Aizheng = Chinese journal of cancer* 2009, **28**(11):1132-1137.
