# Supplementary figures and images for: Identifying optimal candidates for induction chemotherapy among stage II–IVa nasopharyngeal carcinoma based on pretreatment Epstein–Barr virus DNA and nodal maximal standard uptake values of [18F]‐fluorodeoxyglucose positron emission tomography
Source: Cancer Med. 2020 Oct 9;9(23):8852–63. doi: 10.1002/cam4.3500 (PMC7724500; doi:10.1002/cam4.3500)

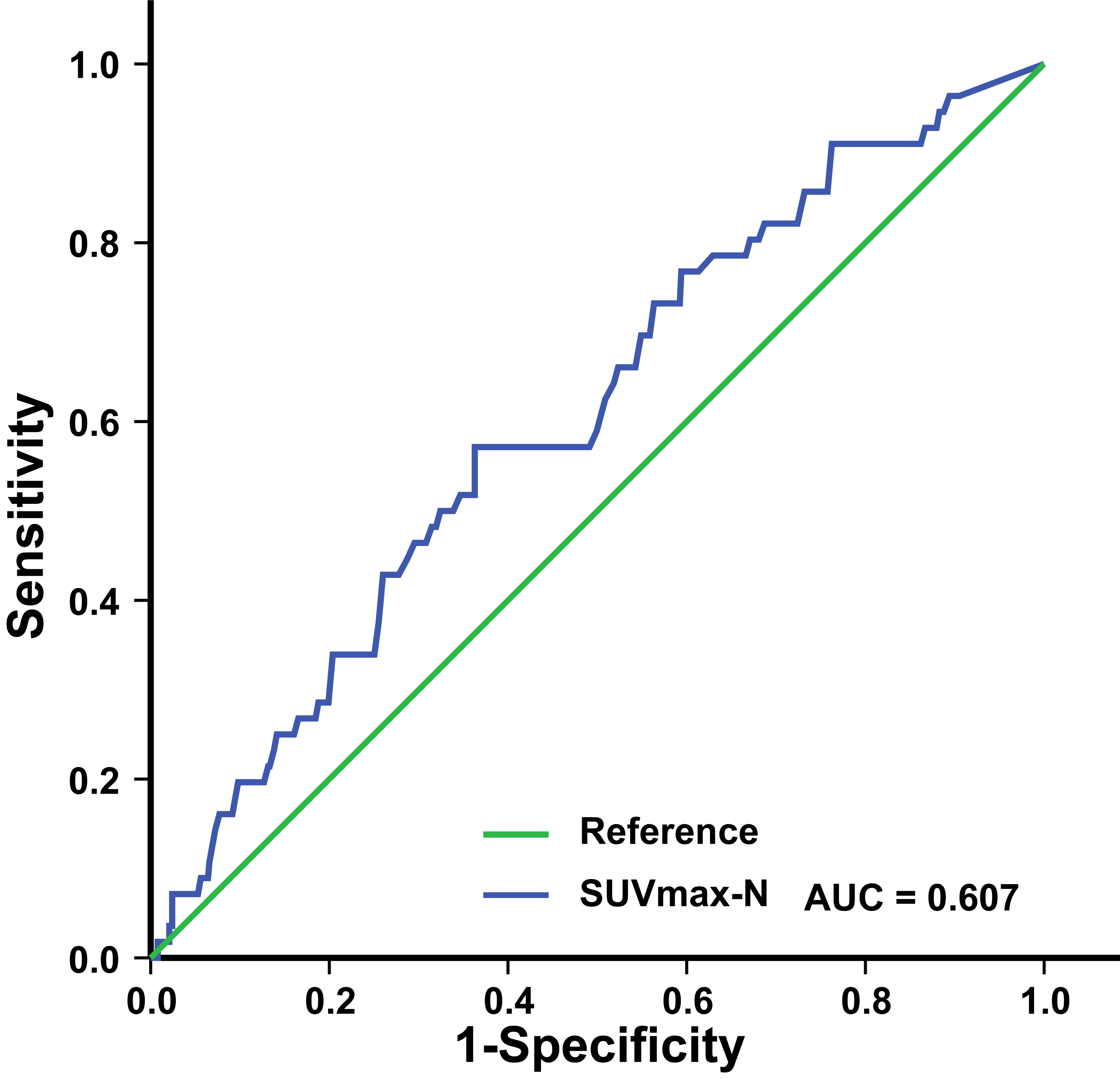

Supplement: Supplementary file 2 — Fig S1 [file CAM4-9-8852-s002.tif]

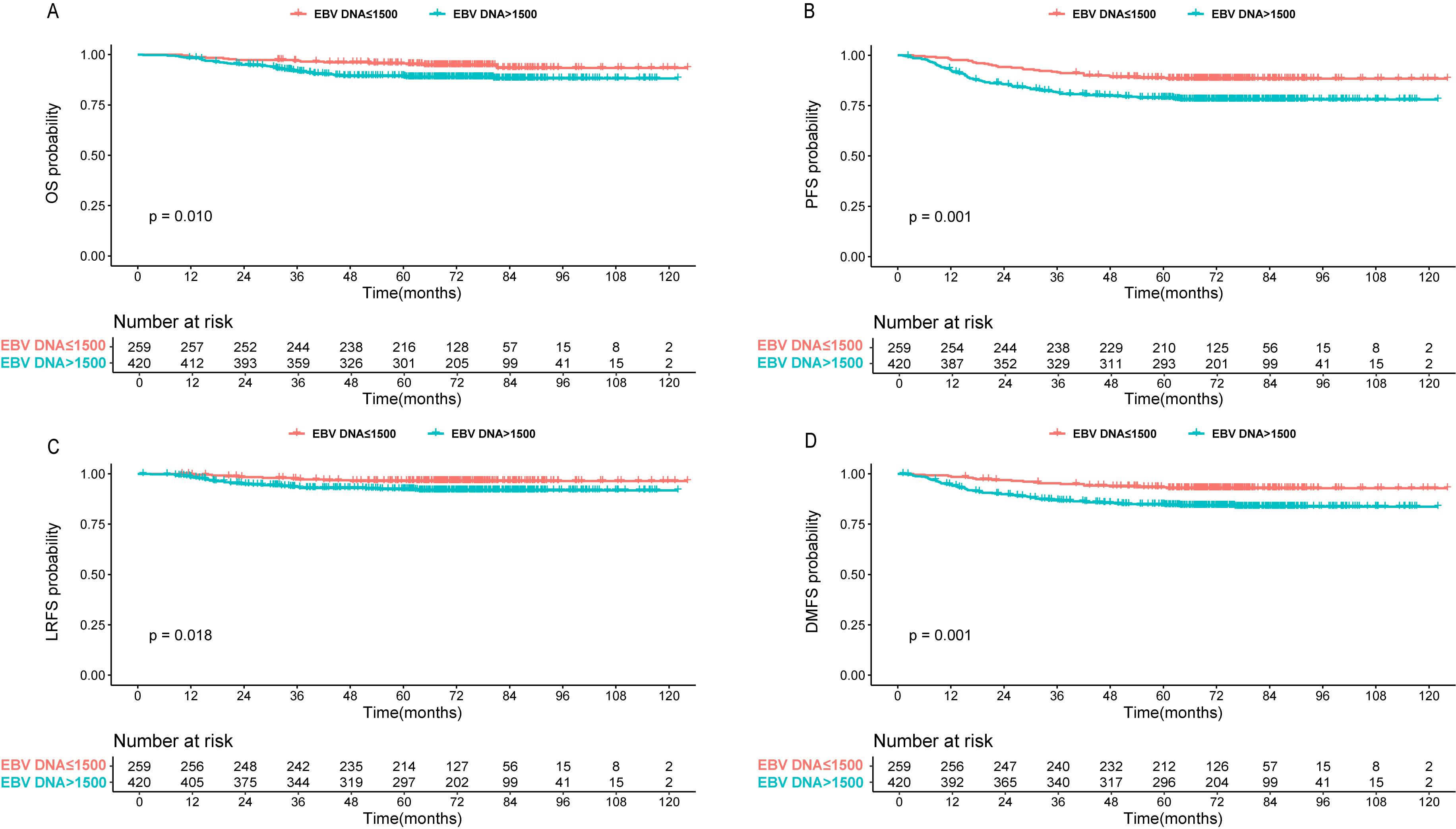

Supplement: Supplementary file 3 — Fig S2 [file CAM4-9-8852-s003.tif]

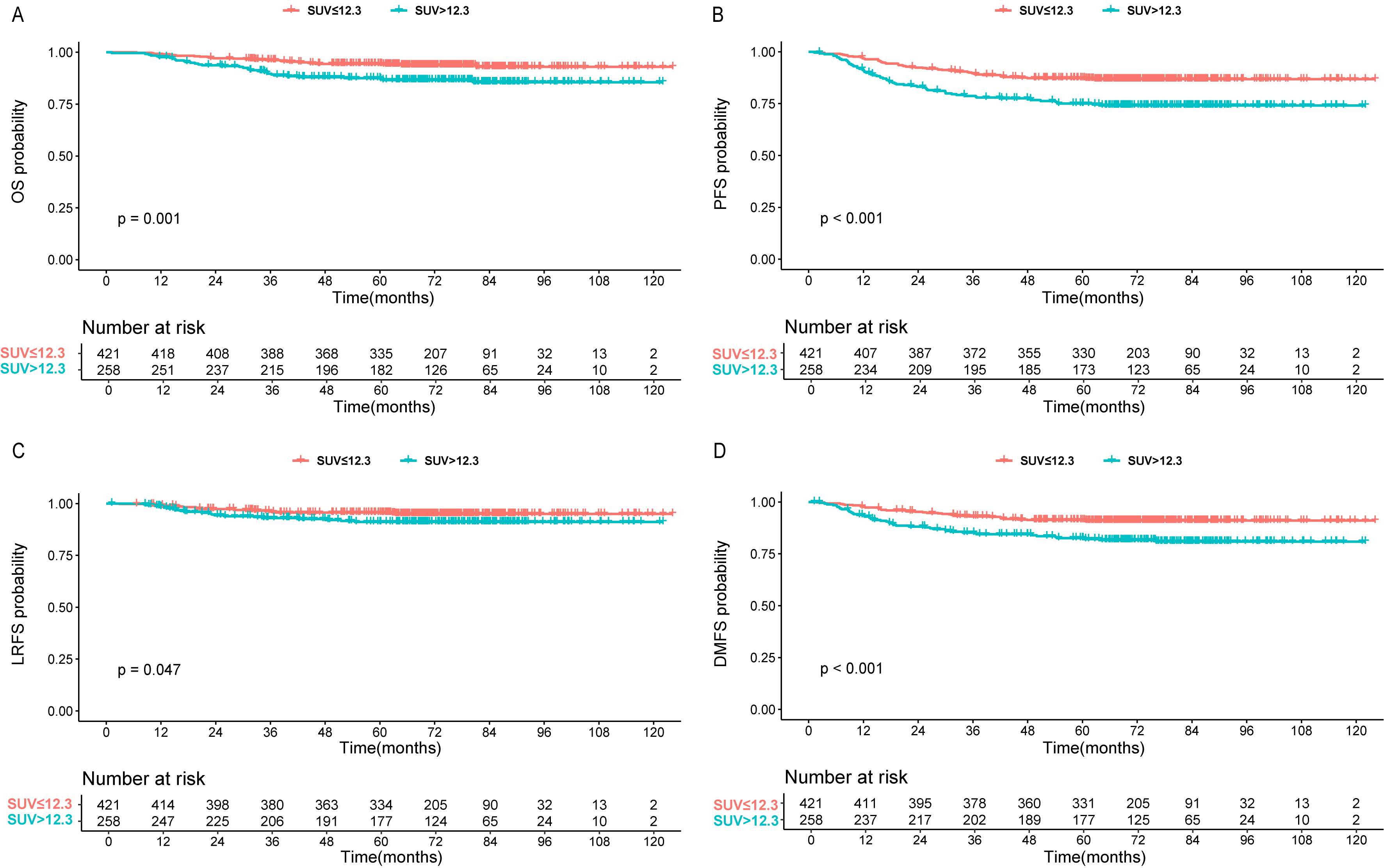

Supplement: Supplementary file 4 — Fig S3 [file CAM4-9-8852-s004.tif]

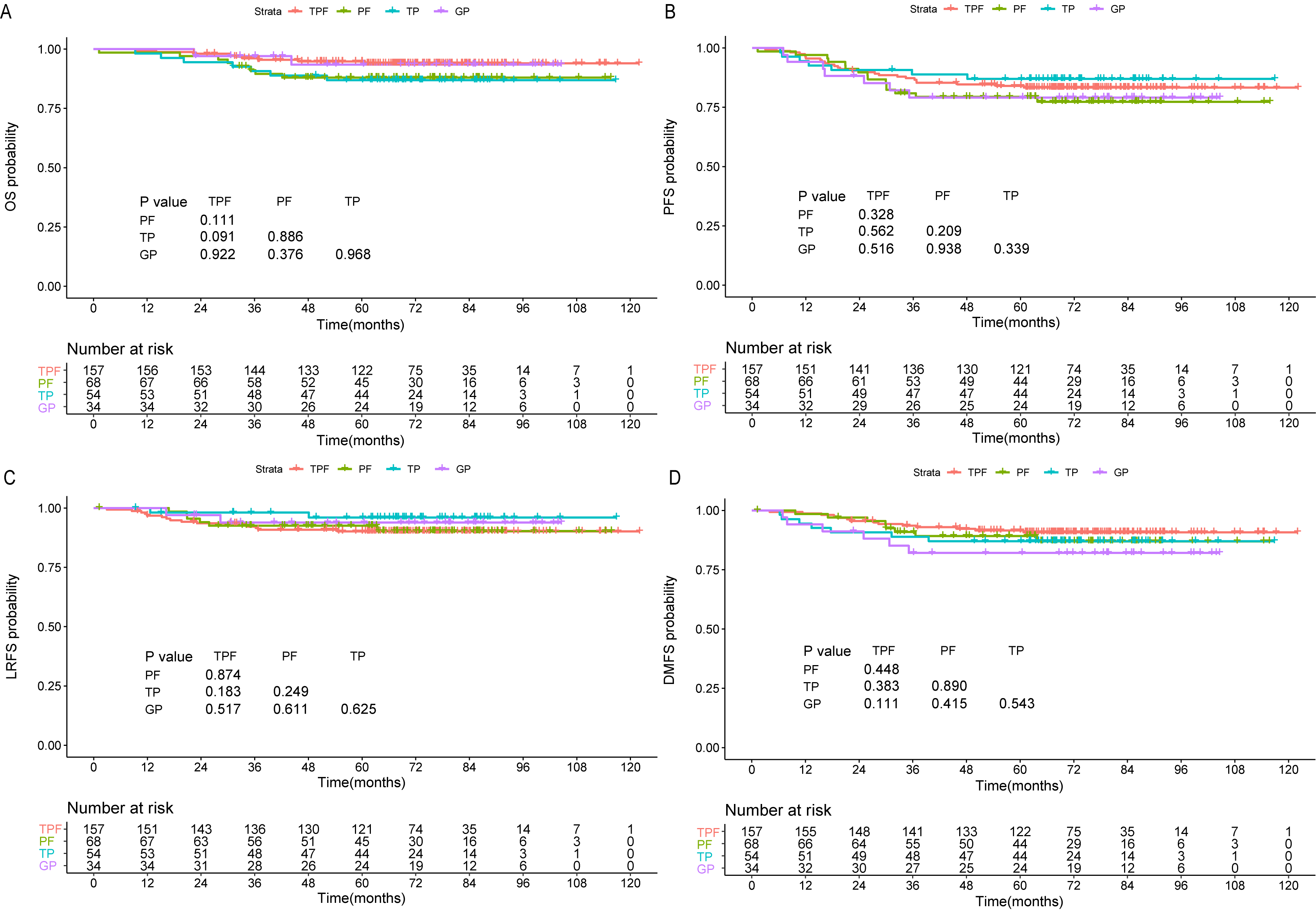

Supplement: Supplementary file 5 — Fig S4 [file CAM4-9-8852-s005.tif]
