# Supplementary material for: Identifying optimal candidates for induction chemotherapy among stage II–IVa nasopharyngeal carcinoma based on pretreatment Epstein–Barr virus DNA and nodal maximal standard uptake values of [18F]‐fluorodeoxyglucose positron emission tomography
Source: Cancer Med. 2020 Oct 9;9(23):8852–63. doi: 10.1002/cam4.3500 (PMC7724500; doi:10.1002/cam4.3500)
Supplement: Supplementary file 6 — Table S1 [file CAM4-9-8852-s006.docx]

Table S1 Clinical Characteristics of Patients in Low- and High-Risk Subgroups

|  | Low-risk patients  n (%) | | | High-risk patients n (%) | | |
| --- | --- | --- | --- | --- | --- | --- |
| Characteristic | CCRT alone | CCRT+IC | *P value* | CCRT alone | CCRT+IC | *P value* |
| **Total** | 273 (100.0%) (100.0%) | 196 (100%) (100.0%) (100.0%) |  | 93 (100.0%) (100.0%) | 117 (100.0%) (100.0%) |  |
| **Age, y** |  |  | 0.190 |  |  | 0.127 |
| ≤46 | 150 (54.9%) | 95 (48.5%) |  | 39 (41.9%) | 62 (53.0%) |  |
| >46 | 123 (45.1%) | 101 (51.5%) |  | 54 (58.1%) | 55 (47.0%) |  |
| **Gender** |  |  | 0.822 |  |  | 0.868 |
| Female | 62 (22.7%) | 42 (21.4%) |  | 21 (22.6%) | 25 (21.4%) |  |
| Male | 211 (77.3%) | 154 (78.6%) |  | 72 (77.4%) | 92 (78.6%) |  |
| **Smoking history** |  |  | 0.924 |  |  | 0.777 |
| No | 162 (59.3%) | 115 (58.7%) |  | 55 (59.1%) | 72 (61.5%) |  |
| Yes | 111 (40.7%) | 81 (41.3%) |  | 38 (40.9%) | 45 (38.5%) |  |
| **Family history of NPC** | |  | 0.733 |  |  | 0.999 |
| No | 252 (92.3%) | 179 (91.3%) |  | 82 (88.2%) | 104 (88.9%) |  |
| Yes | 21 (7.7%) | 17 (8.7%) |  | 11 (11.8%) | 13 (11.1%) |  |
| **Overall stage** |  |  | <0.001 |  |  | 0.013 |
| II | 30 (11.0%) | 7 (3.6%) |  | 4 (4.3%) | 1 (0.9%) |  |
| III | 188 (68.9%) | 96 (49.0%) |  | 51 (54.8%) | 47 (40.2%) |  |
| IV | 55 (20.1%) | 93 (47.4%) |  | 38 (40.9%) | 69 (59.0%) |  |
| **T stage^a^** |  |  | <0.001 |  |  | 0.209 |
| T1 | 24 (8.8%) | 4 (2.0%) |  | 6 (6.5%) | 2 (1.7%) |  |
| T2 | 48 (17.6%) | 24 (12.2%) |  | 14 (15.1%) | 15 (12.8%) |  |
| T3 | 165 (60.4%) | 94 (48.0%) |  | 50 (53.8%) | 61 (52.1%) |  |
| T4 | 36 (13.2%) | 74 (37.8%) |  | 23 (24.7%) | 39 (33.3%) |  |
| **N stage^a^** |  |  | 0.079 |  |  | 0.205 |
| N1 | 149 (54.6%) | 90 (45.9%) |  | 23 (24.7%) | 20 (17.1%) |  |
| N2 | 101 (37.0%) | 79 (40.3%) |  | 48 (51.6%) | 58 (49.6%) |  |
| N3 | 23 (8.4%) | 27 (13.8%) |  | 22 (23.7%) | 39 (33.3%) |  |
| **LDH level** |  |  | 0.627 |  |  | 0.054 |
| ≤245 U/L | 262 (96.0%) | 190 (96.9%) |  | 84 (90.3%) | 94 (80.3%) |  |
| >245 U/L | 11 (4.0%) | 6 (3.1%) |  | 9 (9.7%) | 23 (19.7%) |  |
| **SUVmax-N** |  |  | 0.877 |  |  |  |
| ≤12.3 | 244 (89.4%) | 177 (90.3%) |  |  |  |  |
| >12.3 | 29 (10.6%) | 19 (9.7%) |  | 93 (100%) | 117 (100%) |  |
| **EBV DNA level** | |  | <0.001 |  |  | 0.837 |
| ≤1500 copies/ml | 171 (62.6%) | 88 (44.9%) |  |  |  |  |
| 1500-4000 copies/ml | 37 (13.6%) | 33 (16.8%) |  | 11 (11.8%) | 15 (12.8%) |  |
| >4000 | 65 (23.8%) | 75 (38.3%) |  | 82 (88.2%) | 102 (87.2%) |  |
| **IC regimen*** |  |  |  |  |  |  |
| TPF |  | 96 (49.0%) |  |  | 61 (52.1%) |  |
| PF |  | 44 (22.4%) |  |  | 24 (20.5%) |  |
| TP |  | 38 (19.4%) |  |  | 16 (13.7%) |  |
| GP |  | 18 (9.2%) |  |  | 16 (13.7%) |  |
| **IC cycle*** |  |  |  |  |  |  |
| 2 cycles |  | 105 (53.6%) |  |  | 59 (50.4%) |  |
| 3 cycles |  | 66 (33.7%) |  |  | 45 (38.5%) |  |
| 4 cycles |  | 25 (12.8%) |  |  | 13 (11.1%) |  |

Abbreviations: CCRT, concurrent chemoradiotherapy; IC, induction chemotherapy; EBV, Epstein–Barr virus; TPF cisplatin plus docetaxel plus 5-fluorouracil; TP, cisplatin plus docetaxel; PF, cisplatin plus 5-fluorouracil; GP, cisplatin plus gemcitabine

**^a^** According to the 8th edition of the UICC/AJCC staging system.

* Only patients treated with IC+CCRT were analysis.

*P* values were calculated by a χ^2^ test.
