# Supplementary material for: Identifying optimal candidates for induction chemotherapy among stage II–IVa nasopharyngeal carcinoma based on pretreatment Epstein–Barr virus DNA and nodal maximal standard uptake values of [18F]‐fluorodeoxyglucose positron emission tomography
Source: Cancer Med. 2020 Oct 9;9(23):8852–63. doi: 10.1002/cam4.3500 (PMC7724500; doi:10.1002/cam4.3500)
Supplement: Supplementary file 7 — Table S2 [file CAM4-9-8852-s007.docx]

Table S2. Acute toxicities of patients in CCRT and IC+CCRT groups

|  | CCRT alone | CCRT+IC | P value |
| --- | --- | --- | --- |
| **Leukocytopenia** |  |  |  |
| G0-2 | 306 (83.7%) | 210 (67.2%) | <0.001 |
| G3-4 | 60 (16.3%) | 103 (32.8%) |  |
| **Neutropenia** |  |  |  |
| G0-2 | 324 (88.4%) | 189 (60.5%) | <0.001 |
| G3-4 | 42 (11.6%) | 124 (39.5%) |  |
| **Anemia** |  |  |  |
| G0-2 | 361 (98.6%) | 307 (98.2%) | 0.762 |
| G3-4 | 5 (1.4%) | 6 (1.8%) |  |
| **Thrombocytopenia** |  |  |  |
| G0-2 | 359 (98.0%) | 307 (98.1%) | 1.000 |
| G3-4 | 7 (2.0%) | 6 (1.9%) |  |
| **Hepatotoxicity** |  |  |  |
| G0-2 | 362 (98.8%) | 308 (98.5%) | 0.739* |
| G3-4 | 4 (1.2%) | 5 (1.5%) |  |
| **Nephrotoxicity** |  |  |  |
| G0-2 | 366 (100%) | 312 (99.7%) | 0.461* |
| G3-4 | 0 (0.0%) | 1 (0.3%) |  |

The P value was calculated using the χ^2^ test or Fisher’s exact test (*).
